# Complete genome sequence of *Oceanithermus profundus* type strain (506^T^)

**DOI:** 10.4056/sigs.1734292

**Published:** 2011-04-29

**Authors:** Amrita Pati, Xiaojing Zhang, Alla Lapidus, Matt Nolan, Susan Lucas, Tijana Glavina Del Rio, Hope Tice, Jan-Fang Cheng, Roxane Tapia, Cliff Han, Lynne Goodwin, Sam Pitluck, Konstantinos Liolios, Ioanna Pagani, Natalia Ivanova, Konstantinos Mavromatis, Amy Chen, Krishna Palaniappan, Loren Hauser, Cynthia D. Jeffries, Evelyne-Marie Brambilla, Alina Röhl, Romano Mwirichia, Manfred Rohde, Brian J. Tindall, Johannes Sikorski, Reinhard Wirth, Markus Göker, Tanja Woyke, John C. Detter, James Bristow, Jonathan A. Eisen, Victor Markowitz, Philip Hugenholtz, Nikos C. Kyrpides, Hans-Peter Klenk, Miriam Land

**Affiliations:** 1 DOE Joint Genome Institute, Walnut Creek, California, USA; 2 Los Alamos National Laboratory, Bioscience Division, Los Alamos, New Mexico, USA; 3 Biological Data Management and Technology Center, Lawrence Berkeley National Laboratory, Berkeley, California, USA; 4 Oak Ridge National Laboratory, Oak Ridge, Tennessee, USA; 5 DSMZ - German Collection of Microorganisms and Cell Cultures GmbH, Braunschweig, Germany; 6 University of Regensburg, Microbiology – Archaeenzentrum, Regensburg, Germany; 7 Jomo Kenyatta University of Agriculture and Technology, Nairobi, Kenya; 8 HZI – Helmholtz Centre for Infection Research, Braunschweig, Germany; 9 University of California Davis Genome Center, Davis, California, USA; 10 Australian Centre for Ecogenomics, School of Chemistry and Molecular Biosciences, The University of Queensland, Brisbane, Australia

**Keywords:** microaerophilic, non-motile, Gram-negative, nitrate-reducing, moderate thermophilic, neutrophilic, chemolithoheterotrophic, hydrothermal vent, *Thermaceae*, GEBA

## Abstract

*Oceanithermus profundus* Miroshnichenko *et al.* 2003 is the type species of the genus *Oceanithermus*, which belongs to the family *Thermaceae*. The genus currently comprises two species whose members are thermophilic and are able to reduce sulfur compounds and nitrite. The organism is adapted to the salinity of sea water, is able to utilize a broad range of carbohydrates, some proteinaceous substrates, organic acids and alcohols. This is the first completed genome sequence of a member of the genus *Oceanithermus* and the fourth sequence from the family *Thermaceae*. The 2,439,291 bp long genome with its 2,391 protein-coding and 54 RNA genes consists of one chromosome and a 135,351 bp long plasmid, and is a part of the *** G****enomic* *** E****ncyclopedia of* *** B****acteria and* *** A****rchaea * project.

## Introduction

Strain 506^T^ (DSM 14977 = NBRC 100410 = VKM B-2274) is the type strain of *Oceanithermus profundus*, which is the type species of the genus *Oceanithermus* [1] of the family *Thermaceae* [2]. Together with *O. desulfurans*, there are currently two species placed in the genus [1,3]. The generic name derives from the Latin noun oceanus, meaning *ocean* and the Neo-Latin masc. substantive (from Gr. adj. *thermos*) *thermus* which means *hot*. Therefore, the name *Oceanithermus* refers to warmth-loving organisms living in the ocean. The species epithet is derived from the Latin adjective *profundus* meaning *deep*, which means pertaining to the abyss, pertaining to the depths of the ocean [1]. Strain 506^T^ was first isolated from samples of hydrothermal fluids and chimneys collected at the 13ºN hydrothermal vent field on the East Pacific Rise at a depth of 2600 m [1]. There are no further cultivated strains of this species known. The other member of the genus, *O. desulfurans,* is a thermophilic, sulfur-reducing bacterium isolated from a sulfide chimney in Suiyo Seamount, in the Western Pacific [3]. Here we present a summary classification and a set of features for *O. profundus* 506^T^, together with the description of the complete genomic sequencing and annotation.

## Classification and features

A representative genomic 16S rRNA sequence of strain 506^T^ was compared using NCBI BLAST under default settings (e.g., considering only the high-scoring segment pairs (HSPs) from the best 250 hits) with the most recent release of the Greengenes database [4] and the relative frequencies, weighted by BLAST scores, of taxa and keywords (reduced to their stem) [5] were determined. The five most frequent genera were *Thermus* (52.0%), *Meiothermus* (37.0%), *Oceanithermus* (7.6%), *Marinithermus* (2.0%) and *Vulcanithermus* (1.4%) (156 hits in total). Regarding the four hits to sequences from members of the species, the average identity within HSPs was 99.6%, whereas the average coverage by HSPs was 94.8%. Regarding the two hits to sequences from other members of the genus, the average identity within HSPs was 99.3%, whereas the average coverage by HSPs was 91.0%. Among all other species, the one yielding the highest score was *O. desulfurans*, which corresponded to an identity of 99.3% and an HSP coverage of 91.0%. The highest-scoring environmental sequence was EU555123 ('Microbial Sulfide Hydrothermal Vent Field Juan de Fuca Ridge Dudley hydrothermal vent clone 4132B16'), which showed an identity of 99.1% and an HSP coverage of 98.0%. The five most frequent keywords within the labels of environmental samples which yielded hits were 'spring' (8.2%), 'hot' (6.2%), 'microbi' (4.5%), 'geochem, nation, park, yellowston' (2.8%) and 'hydrotherm/vent' (2.5%) (94 hits in total). The five most frequent keywords within the labels of environmental samples which yielded hits of a higher score than the highest scoring species were 'hydrotherm/vent' (12.2%), 'field, microbi, ridg' (6.1%), 'fluid' (5.9%), 'dudlei, fuca, juan, sulfid' (3.1%) and 'degre, east, north, ocean, pacif, rise' (3.0%) (3 hits in total). These 16S BLAST results are a confirmation of the kind of environment from which the living strain was isolated and therefore fits the description of the isolate.

**Figure 1:**
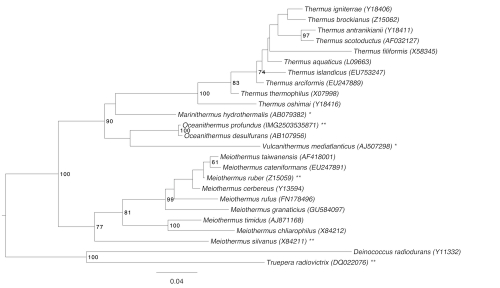
Phylogenetic tree highlighting the position of *O. profundus* relative to the other type strains within the family *Thermaceae*. The tree was inferred from 1,420 aligned characters [6,7] of the 16S rRNA gene sequence under the maximum likelihood criterion [8]. Rooting was initially done using the midpoint method [9] and then checked for its accordance with the current taxonomy (see Table 1) and rooted accordingly. The branches are scaled in terms of the expected number of substitutions per site. Numbers to the right of bifurcations are support values from 1,000 bootstrap replicates [10] if larger than 60%. Lineages with type strain genome sequencing projects that are registered in GOLD [11] but remain unpublished are labeled with one asterisk, published genomes with two asterisks [12-14].

**Figure 1** shows the phylogenetic neighborhood of *O. profundus* in a 16S rRNA based tree. The sequences of the two identical 16S rRNA gene copies in the genome differ by one nucleotide from the previously published 16S rRNA sequence (AJ430586).

**Figure 2:**
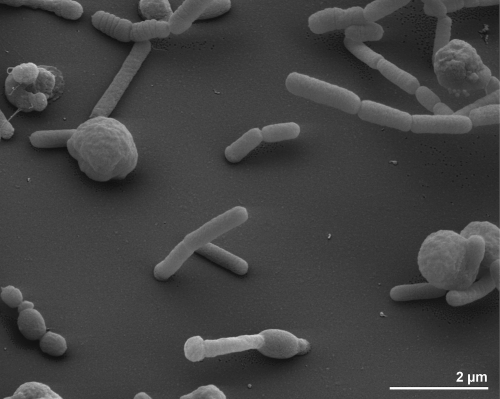
Scanning electron micrograph of *O. profundus* 506^T^

The cells of *O. profundus* are described as non-motile, rod-shaped, 0.5 – 0.7 µm in diameter and of various lengths (Figure 2). When grown on proteinaceous substrates, old cultures of *O. profundus* form filaments and large spheres resembling the ‘rotund bodies’ typical of aged cells of *Thermus* species [1,15]. The organism is Gram-negative and non spore-forming (Table 1).

**Table 1:** Classification and general features of *O. profundus* 506^T^ according to the MIGS recommendations [16].

**MIGS ID**	**Property**	**Term**	**Evidence code**
	Current classification	Domain *Bacteria*	TAS [17]
		Phylum “*Deinococcus-Thermus”*	TAS [18,19]
		Class *Deinococci*	TAS [20,21]
		Order *Thermales*	TAS [21,22]
		Family *Thermaceae*	TAS [21,23]
		Genus *Oceanithermus*	TAS [1]
		Species *Oceanithermus profundus*	TAS [1]
		Type strain 506	TAS [1]
	Gram stain	negative	TAS [1]
	Cell shape	rod-shaped	TAS [1]
	Motility	non-motile	TAS [1]
	Sporulation	none	TAS [1]
	Temperature range	40-68ºC	TAS [1]
	Optimum temperature	60°C	TAS [1]
	Salinity	1%-5%, optimum 3% NaCl	TAS [1]
MIGS-22	Oxygen requirement	microaerophile	TAS [1]
	Carbon source	carbohydrates	TAS [1]
	Energy metabolism	chemoorganoheterotroph, lithoheterotroph, organotroph	TAS [1]
MIGS-6	Habitat	deep sea, hydrothermal vent, marine	TAS [1]
MIGS-15	Biotic relationship	free-living	TAS [1]
MIGS-14	Pathogenicity	none	NAS
	Biosafety level	1	NAS [24]
	Isolation	deep-sea hot vent	TAS [1]
MIGS-4	Geographic location	East Pacific Rise	TAS [1]
MIGS-5	Sample collection time	1999	TAS [1]
MIGS-4.1	Latitude	12.8	TAS [1]
MIGS-4.2	Longitude	103.93	TAS [1]
MIGS-4.3	Depth	2,600 m	TAS [1]
MIGS-4.4	Altitude	-2,600 m	NAS

Evidence codes - IDA: Inferred from Direct Assay (first time in publication); TAS: Traceable Author Statement (i.e., a direct report exists in the literature); NAS: Non-traceable Author Statement (i.e., not directly observed for the living, isolated sample, but based on a generally accepted property for the species, or anecdotal evidence). These evidence codes are from of the Gene Ontology project [25] If the evidence code is IDA, then the property was directly observed by one of the authors or an expert mentioned in the acknowledgements.

*O. profundus* is microaerophilic, only being able to grow at oxygen concentrations below 6% [1]. No growth has been observed in an atmosphere of air, either in liquid medium or on plates. In an agar tube containing 5 ml of basal medium supplemented with 2 g sucrose and 1 g tryptone per liter with air in the headspace (10 ml), growth occurs in a zone located 20 mm below the agar/air interface [1]. Alternatively, the organism grows anaerobically using nitrate as the electron acceptor. *O. profundus* grows within a temperature range of 40-68ºC, optimal growth being observed at 60ºC. At 60ºC, it grows between pH 5.5 and 8.4, with an optimum around 7.5 [1]. Strain 506^T^ grows at NaCl concentrations ranging from 10 to 50 g/l, with an optimum at 30 g/1 [1]. The organism is oxidase- and catalase positive and is able to utilize a wide spectrum of carbohydrates in the presence of either nitrate or oxygen [1]. The highest cell yield is observed in the presence of nitrate with fructose, maltose, sucrose, trehalose, galactose, rhamnose or xylose. Glucose, lactose and starch are utilized, but no growth has been reported with ribose, galactose, arabinose, dextrin or cellobiose [1]. Acetate and propionate are produced during growth with sucrose as a growth substrate and nitrate as the electron acceptor. Nitrite is the only product of denitrification [1]. *O. profundus* grows well with complex proteinaceous substrates such as beef extract, tryptone or papaic digest of soybean (1-1.5 g/l). However, growth is strongly inhibited by higher concentrations of these substrates [1]. The isolate does not grow with Casamino acids or yeast extract as sole sources of carbon and energy, though 100 mg/l yeast extract is required for growth [1]. *O. profundus* is able to utilize acetate, pyruvate and propionate as growth substrates. It also grows with methanol, ethanol and mannitol, though the cell yield is lower [1]. *O. profundus* is able to grow lithoheterotrophically using molecular hydrogen as the energy source, yeast extract as the carbon source and nitrate as the electron acceptor. Other electron acceptors (sulfate, elemental sulfur, thiosulfate and nitrite) do not support growth, regardless of growth substrate [1]. Detailed studies on the metabolism of maltose, acetate, pyruvate, and hydrogen have been undertaken by Fedosov *et al.* [26].

### Chemotaxonomy

The polar lipid pattern of strain 506^T^ comprises three phospholipids, whereas glycolipids have not been detected [1]. This differentiates the organism from members of the genera *Vulcanithermus, Rhabdothermus*, *Thermus* and *Meiothermus*, where phospholipids and glycolipids have both been detected [27,28]. It should be noted that the major phospholipid detected in *O. profundus* has the same R_f_ and staining behavior as the 2′-O-(1, 2-diacyl-*sn*-glycero-3-phospho)–3′-O-(α-N-acetyl-glucosaminyl)-N-glyceroyl alkylamine reported to occur in members of the genera *Meiothermus* and *Thermus* [29]. On the basis of R_f_ value and staining behavior this lipid also appears to be present in members of the genera *Vulcanithermus* and *Rhabdothermus*, which also synthesize glycolipids [30,31]. Although members of the genus *Deinococcus* may also produce glycolipids in addition to a novel series of phosphoglycolipids [32,33] the latter are absent in members of the genera *Thermus* and *Meiothermus*. The absence of glycolipids was one of the arguments for Miroshnichenko *et al*. for placing strain 506^T^ in a new genus [1].

Menaquinones are the sole respiratory lipoquinones detected, with MK-8 predominating (95%) and MK-9 being present in smaller proportions (5%) [1]. The predominance of MK-8 is consistent with reports of MK-8 in members of the genera *Thermus*, *Meiothermus* [34,35], *Marinithermus* [36] *Vulcanithermus, Rhabdothermus, Truepera, Deinobacterium* and *Deinococcus* [30-33,37]. However, the presence of MK-9, albeit at only 5%, appears to be a unique feature of *O. profundus*.

The fatty acids comprise mainly *iso*- and *anteiso*-branched fatty acids though *iso*-unsaturated fatty acids are also present [1]. The major fatty acids are *iso*-C_15:1_ω7 (7.7%), *iso*-C_15:0_ (33.2%), *iso*-C_16:1_ω8 (2.6 *iso*-C_16:0_ (3.3%), *iso*-C_17:1_ω7c (18.8%), *iso*-C_17:0_ (12.3%), *anteiso*-C_15:0_ (5.1%) and *anteiso*-C_17:0_ (5.4%) [1]. The presence of *iso*- and *anteiso*-branched fatty acids is a feature of members of the genera *Deinococcus*, *Thermus*, *Meiothermus, Vulcanithermus, Rhabdothermus* and *Marinithermus* [27,28,30-34,37]. The presence of unsaturated branched-chain fatty acids is a distinctive feature of members of the genera *Oceanithermus, Vulcanithermus* and *Rhabdothermus* within the family *Thermaceae*. The unsaturated fatty acid content of the isolate is also higher (33-37%) as compared to the closest relative *O. desulfurans* (18%) [3].

## Genome sequencing and annotation

### Genome project history

This organism was selected for sequencing on the basis of its phylogenetic position [38] and is part of the *** G****enomic* *** E****ncyclopedia of* *** B****acteria and* *** A****rchaea * project [39]. The genome project is deposited in the Genome On Line Database [11] and the complete genome sequence is deposited in GenBank. Sequencing, finishing and annotation were performed by the DOE Joint Genome Institute (JGI). A summary of the project information is shown in Table 2.

**Table 2:** Genome sequencing project information

**MIGS ID**	**Property**	**Term**
MIGS-31	Finishing quality	Finished
MIGS-28	Libraries used	Three genomic libraries: one 454 pyrosequence standard library, one 454 PE library (17 kb insert size), one Illumina library
MIGS-29	Sequencing platforms	Illumina GAii, 454 GS FLX Titanium
MIGS-31.2	Sequencing coverage	85.5 × Illumina; 197.3 × pyrosequence
MIGS-30	Assemblers	Newbler version 2.3-PreRelease-8-23-2009, Velvet, phrap
MIGS-32	Gene calling method	Prodigal 1.4, GenePRIMP
	INSDC ID	CP002361 chromosome CP002362 plasmid OCEPR01
	Genbank Date of Release	December 7, 2010
	GOLD ID	Gc01553
	NCBI project ID	40223
	Database: IMG-GEBA	2503508010
MIGS-13	Source material identifier	DSM 14977
	Project relevance	Tree of Life, GEBA

### Growth conditions and DNA isolation

*O. profundus* strain 506^T^, DSM 14977, was grown anaerobically in DSMZ medium 975 (*Oceanithermus profundus* medium) [40] at 60°C. DNA was isolated from 0.5-1 g of cell paste using Jetflex Genomic DNA Purification Kit following the standard protocol as recommended by the manufacturer, but with an additional proteinase K (20 µl) digestion for 45 min at 58°C. DNA is available through the DNA Bank Network [41].

### Genome sequencing and assembly

The genome was sequenced using a combination of Illumina and 454 sequencing platforms. All general aspects of library construction and sequencing can be found at the JGI website [42]. Pyrosequencing reads were assembled using the Newbler assembler version 2.3-PreRelease-8-23-2009 (Roche). The initial Newbler assembly, consisting of nine contigs in four scaffolds, was converted into a phrap assembly by [43] making fake reads from the consensus, to collect the read pairs in the 454 paired end library. Illumina GAii sequencing data (208 Mb) was assembled with Velvet [44] and the consensus sequences were shredded into 1.5 kb overlapped fake reads and assembled together with the 454 data. The 454 draft assembly was based on 306.1 Mb 454 draft data and all of the 454 paired end data. Newbler parameters are -consed -a 50 -l 350 -g -m -ml 20. The Phred/Phrap/Consed software package [43] was used for sequence assembly and quality assessment in the subsequent finishing process. After the shotgun stage, reads were assembled with parallel phrap (High Performance Software, LLC). Possible mis-assemblies were corrected with gapResolution [42], Dupfinisher, or sequencing cloned bridging PCR fragments with subcloning or transposon bombing (Epicentre Biotechnologies, Madison, WI) [45]. Gaps between contigs were closed by editing in Consed, by PCR and by Bubble PCR primer walks (J.-F.Chang, unpublished). A total of 177 additional reactions were necessary to close gaps and to raise the quality of the finished sequence. Illumina reads were also used to correct potential base errors and increase consensus quality using a software Polisher developed at JGI [46]. The error rate of the completed genome sequence is less than 1 in 100,000. Together, the combination of the Illumina and 454 sequencing platforms provided 282.8 × coverage of the genome. The final assembly contained 1,258,374 pyrosequence and 5,792,221 Illumina reads.

### Genome annotation

Genes were identified using Prodigal [47] as part of the Oak Ridge National Laboratory genome annotation pipeline, followed by a round of manual curation using the JGI GenePRIMP pipeline [48]. The predicted CDSs were translated and used to search the National Center for Biotechnology Information (NCBI) nonredundant database, UniProt, TIGR-Fam, Pfam, PRIAM, KEGG, COG, and InterPro databases. Additional gene prediction analysis and functional annotation was performed within the Integrated Microbial Genomes - Expert Review (IMG-ER) [49].

## Genome properties

**Figure 3:**
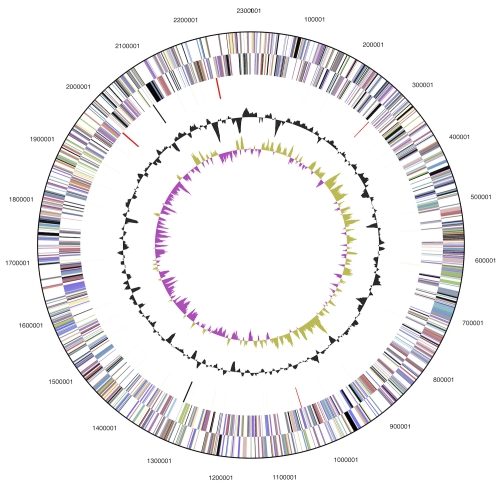
Graphical circular map of chromosome (map of plasmid not shown). From outside to the center: Genes on forward strand (color by COG categories), Genes on reverse strand (color by COG categories), RNA genes (tRNAs green, rRNAs red, other RNAs black), GC content, GC skew.

The genome consists of a 2,303,940 bp long chromosome with a G+C content of 70% and a 135,351 bp plasmid with a G+C content of 66% (Table 3 and Figure 3). Of the 2,445 genes predicted, 2,391 were protein-coding genes, and 54 RNAs; 18 pseudogenes were also identified. The majority of the protein-coding genes (69.9%) were assigned with a putative function while the remaining ones were annotated as hypothetical proteins. The distribution of genes into COGs functional categories is presented in Table 4.

**Table 3:** Genome Statistics

**Attribute**	**Value**	**% of Total**
Genome size (bp)	2,439,291	100.00%
DNA coding region (bp)	2,265,747	92.89%
DNA G+C content (bp)	1,702,985	69.81%
Number of replicons	2	
Extrachromosomal elements	1	
Total genes	2,445	100.00%
RNA genes	54	2.21%
rRNA operons	2	
Protein-coding genes	2,391	97.79%
Pseudo genes	18	0.74%
Genes with function prediction	1,709	69.90%
Genes in paralog clusters	25	1.02%
Genes assigned to COGs	1,772	72.47%
Genes assigned Pfam domains	1,842	75.34%
Genes with signal peptides	615	25.15%
Genes with transmembrane helices	654	26.75%
CRISPR repeats	0	

**Table 4:** Number of genes associated with the general COG functional categories

Code	value	%age	Description
J	150	7.7	Translation, ribosomal structure and biogenesis
A	1	0.0	RNA processing and modification
K	90	4.6	Transcription
L	91	4.7	Replication, recombination and repair
B	1	0.0	Chromatin structure and dynamics
D	27	1.4	Cell cycle control, cell division, chromosome partitioning
Y	0	0.0	Nuclear structure
V	31	1.6	Defense mechanisms
T	80	4.1	Signal transduction mechanisms
M	79	4.1	Cell wall/membrane/envelope biogenesis
N	23	1.2	Cell motility
Z	0	0.0	Cytoskeleton
W	0	0.0	Extracellular structures
U	47	2.4	Intracellular trafficking, secretion, and vesicular transport
O	82	4.2	Posttranslational modification, protein turnover, chaperones
C	154	7.9	Energy production and conversion
G	125	6.4	Carbohydrate transport and metabolism
E	203	10.4	Amino acid transport and metabolism
F	72	3.7	Nucleotide transport and metabolism
H	93	4.8	Coenzyme transport and metabolism
I	66	3.4	Lipid transport and metabolism
P	100	5.1	Inorganic ion transport and metabolism
Q	31	1.6	Secondary metabolites biosynthesis, transport and catabolism
R	244	12.5	General function prediction only
S	155	8.0	Function unknown
-	673	27.6	Not in COGs
